# The Prognostic Impact of Intratumoral Aryl Hydrocarbon Receptor in Primary Breast Cancer Depends on the Type of Endocrine Therapy: A Population-Based Cohort Study

**DOI:** 10.3389/fonc.2021.642768

**Published:** 2021-05-20

**Authors:** Helga Tryggvadottir, Emma Sandén, Sofie Björner, Alessandra Bressan, Maria Ygland Rödström, Somayeh Khazaei, Dean P. Edwards, Björn Nodin, Karin Jirström, Karolin Isaksson, Signe Borgquist, Helena Jernström

**Affiliations:** ^1^ Division of Oncology, Department of Clinical Sciences, Lund, Lund University and Skåne University Hospital, Lund, Sweden; ^2^ Department of Molecular & Cellular Biology and Dan L. Duncan Comprehensive Cancer Center, Baylor College of Medicine, Houston, TX, United States; ^3^ Division of Oncology and Therapeutic Pathology, Department of Clinical Sciences, Lund, Lund University and Skåne University Hospital, Lund, Sweden; ^4^ Division of Surgery, Department of Clinical Sciences, Lund, Lund University, Lund, Sweden; ^5^ Department of Surgery, Kristianstad Hospital, Kristianstad, Sweden; ^6^ Department of Oncology, Aarhus University and Aarhus University Hospital, Aarhus, Denmark

**Keywords:** breast cancer, aryl hydrocarbon receptor, intratumoral aromatase, endocrine therapy, polymorphisms, prognosis

## Abstract

The aryl hydrocarbon receptor (AhR) is a master regulator of multiple pathways involved in breast cancer, and influences the estrogen receptor alpha (ER) and aromatase/CYP19A1. The purpose of this study was to elucidate the interplay between intratumoral levels of AhR and aromatase, patient characteristics (including *AhR* and *CYP19A1* genotypes), clinicopathological features, and prognosis in breast cancer patients receiving adjuvant treatments. A prospective cohort of 1116 patients with primary breast cancer in Sweden, included 2002–2012, was followed until June 30^th^ 2019 (median 8.7 years). Tumor‐specific AhR (n=920) and aromatase levels (n=816) were evaluated on tissue microarrays using immunohistochemistry. Associations between cytoplasmatic (AhR^cyt^) and nuclear (AhR^nuc^) AhR levels, intratumoral aromatase, clinicopathological features, and prognosis in different treatment groups were analyzed. Low AhR^cyt^ levels (n=183) and positive intratumoral aromatase (n=69) were associated with estrogen receptor (ER)^–^ status and more aggressive tumors. Genotypes were not associated with their respective protein levels. The functional *AhR* ^Arg554Lys^ GG genotype was associated with recurrence-free survival in switch-therapy (sequential tamoxifen/aromatase inhibitors (AI) or AI/tamoxifen) treated patients (HR_adj_ 0.42; 95% CI 0.22–0.83). High AhR^cyt^ levels were associated with longer recurrence-free survival during the first 10 years of follow-up among tamoxifen-only treated patients (HR_adj_ 0.40; 95% CI 0.23–0.71) compared to low AhR^cyt^ levels, whereas an almost inverse association was seen in patients with switch-therapy (*P*_interaction_=0.023). Intratumoral aromatase had little prognostic impact. These findings warrant confirmation in an independent cohort, preferably in a randomized clinical trial comparing different endocrine regimens. They might also guide the selection of breast cancer patients for clinical trials with selective AhR modulators.

## Introduction

Breast cancer remains an important cause of disease burden and death in women despite novel therapeutic options (1). Along with new treatments, important predictive markers have emerged, guiding the selection of targeted breast cancer therapies (2). However, novel prognostic and predictive tumor markers that can be targeted with new or repurposed treatment choices are urgently needed to minimize both over- and undertreatment. Most breast cancer patients receive adjuvant endocrine therapy, and some up to 10 years following surgery (2). Two potential intratumoral markers that merit further investigation in the adjuvant setting are the master regulator aryl hydrocarbon receptor (AhR) and aromatase, the key enzyme in androgen to estrogen conversion.

We have previously shown that the *AhR* ^Arg554Lys^ polymorphism modifies the relationship between aromatase inhibitor (AI) response and *CYP1A2* (3), which is an important enzyme in estrogen metabolism. AhR is a ligand-activated transcription factor with a wide range of endogenous and exogenous ligands (e.g., toxins, such as dioxin, and several drugs including raloxifene, 4-hydroxy-tamoxifen, in addition to tobacco smoke, and cruciferous vegetables) (4–7). After binding to ligands, the cytoplasmic AhR (AhR^cyt^) translocates to the nucleus and dimerizes with its transcriptional partner AhR nuclear translocator (ARNT), whereby the complex recognizes the dioxin response elements in the promoter of downstream genes that include several *cytochrome P450* (*CYP*) genes (8). These genes include *CYP19A1*, which encodes aromatase, in addition to *CYP1A1*, *CYP1A2*, and *CYP1B1* (9, 10). Therefore, the subcellular AhR localization may be of importance.

The functional *AhR* ^Arg554Lys^ polymorphism influences the mRNA expression level of AhR (11). We have previously reported an association between the same *AhR* ^Arg554Lys^ polymorphism and ER status in breast cancer, and that this polymorphism impacted response to endocrine switch-therapy with sequential tamoxifen/AI or AI/tamoxifen (12). Some groups have reported that various *CYP19A1* genotypes might impact response to AIs (13–16), but this association has been confirmed neither by our group nor others (3, 17).

AhR regulates multiple pathways that might influence all the major stages of carcinogenesis (18). High expression of AhR in breast cancer has been associated with signaling pathways related to metabolism and insulin-like growth factor (IGF) signaling (19). AhR acts as an immunomodulator (8) and is a suggested link between inflammation and breast cancer (20). Several studies have investigated the prognostic impact of AhR expression in breast cancer but with inconsistent results (5, 19, 21–23). A couple of these studies suggested a positive association between AhR levels and good prognosis (5, 22), whereas others indicated a negative prognostic impact (21), that possibly differed between subgroups of patients and according to the intracellular localization of AhR (23).

AhR can be effectively modulated by its ligands, e.g., selective AhR modulators (24), resulting in either agonistic or antagonistic effects on many of the hallmarks of cancer (6). A clinical phase I trial investigating an AhR inhibitor in patients with advanced solid tumors is ongoing (NCT04069026).

The cross-talk between the AhR and the estrogen receptor alpha (ER) plays a major role in signaling processes in female reproductive organs, and it has been shown in *in vivo* models that ligand-activated AhR confers anti-estrogenic effects partly due to lower ER levels in ductal epithelial cells (25). Furthermore, studies have shown AhR-mediated degradation of ER through activation of the proteasome pathway (26, 27). Selective estrogen receptor modulators (SERMs) can act as AhR agonists in some cases (28), such as the active metabolite of tamoxifen, 4-hydroxy-TAM, that modulates the transcriptional activity of AhR (29). Another SERM, raloxifene, induced apoptosis in ER^–^ breast cancer cells (5), which implies that AhR also plays a role in the hormone-independent setting. A small study reported that AhR might induce intratumoral aromatase and thereby stimulate estrogen-dependent breast cancer progression (30). AhR is thus a potential target for new drugs in breast cancer (28, 31).

We hypothesized that high tumor levels of AhR would be associated with good prognosis in breast cancer, but that the prognostic impact might depend on the subcellular AhR localization, treatments, as well as body constitution. We hypothesized that intratumoral AhR and aromatase levels would be associated with each other but not with polymorphisms in their respective genes. The aim was to study associations between intratumoral levels of AhR and aromatase, patient characteristics, including *AhR* and *CYP19A1* genotypes, and clinicopathological features and prognosis in different treatment groups of primary breast cancer patients.

## Materials and Methods

### Study Population

**Figure 1:**
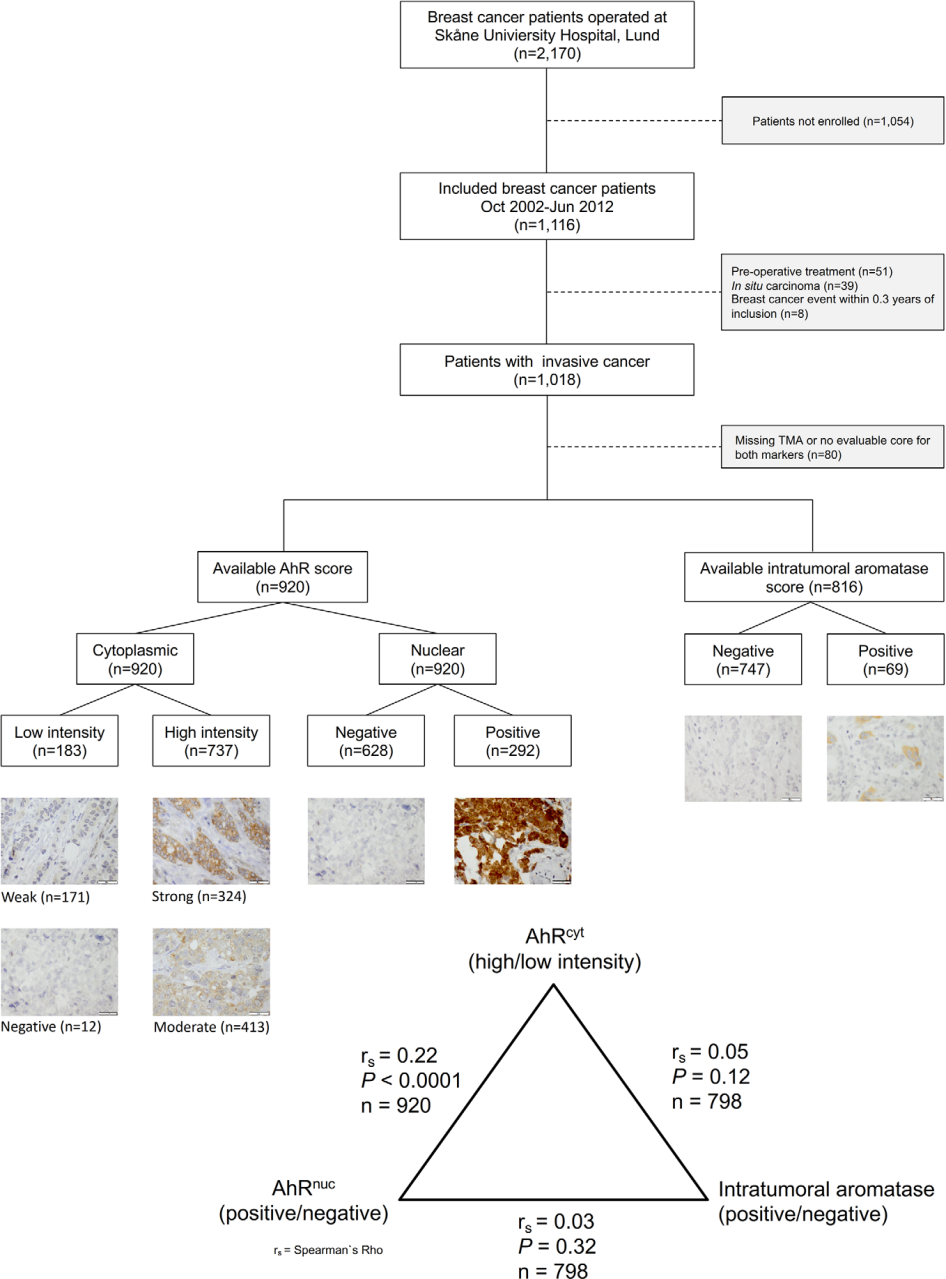
Inclusion flowchart showing the number of included and excluded patients and representative images of AhR and aromatase staining intensities (40×). The bar represents 20 µm.

Primary breast cancer patients in Lund, Sweden were invited to participate in an ongoing prospective study, the Breast Cancer (BC) blood study. Patients with any previous breast cancer or another cancer diagnosis within 10 years were not eligible for participation. Between October 2002 and June 2012, 1116 patients were enrolled. After excluding 51 patients who had received preoperative treatment, 39 patients with *in situ* carcinoma and eight patients with early recurrences within 0.3 years, 1018 patients with invasive breast cancer remained. A flowchart of the selection criteria is presented in **Figure 1**.

Preoperatively, the patients completed a questionnaire as previously described (32). Blood samples were obtained, and body measurements were taken by a trained research nurse. Body mass index (BMI) was calculated, and the cut-off for overweight was set to ≥25 kg/m^2^ according to the WHO classification (33). The waist-to-hip ratio (WHR) was calculated, and the cut-off for central obesity was set to >0.85 since WHO recommends that a woman’s WHR is ≤0.85 (34). Breast size was measured with plastic cups, as described previously (35). Clinical information, including medication use, was retrieved from medical records, and combined with information from questionnaires.

Information on clinical tumor markers, such as ER and progesterone receptor (PR) expression (cut-off at >10% positively stained nuclei according to current clinical routine in Sweden), was collected from pathology reports. HER2 status (amplified/non-amplified) was incorporated into a clinical routine in November 2005 and was available for most tumors after that date. Clinical HER2 status was supplemented with retrospective HER2 analysis by gene protein assay on tissue microarrays (TMA) (36). Information on histological type and grade, invasive tumor size, and axillary node involvement (ALNI) was retrieved from the patient charts and pathology reports.

Patients were followed until June 30^th^, 2019. Information on survival and breast cancer events was obtained from the Swedish Population Registry, the Regional Tumor Registry, pathology reports, and patient charts. Local or regional recurrences, contralateral cancers, or distant metastasis and death due to any cause were considered as endpoints in recurrence-free survival (RFS) analyses. For analyses of distant metastasis-free survival (DMFS), both distant metastasis and death due to any cause were used as an endpoint. Patients who emigrated were followed until the first breast cancer event, if occurring before emigration, and otherwise until the last follow-up before emigration. For all patients living in Sweden, information on subsequent death was obtained from the Swedish Population Registry. Patients without events were censored at the time of the last follow-up before emigration. Other patients were censored at the time of the last follow-up by June 30^th^, 2019.

Adjuvant breast cancer treatment was administered according to clinical recommendations and was only considered before the first breast cancer event. In patients without any breast cancer events, treatments were recorded until the last follow-up or death before July 1^st^, 2019. Written informed consents were obtained from all participating patients, and the study was approved by the local ethics committee at Lund University (Dnr 75-02, Dnr 37-08, Dnr 658-09, Dnr 58-12, Dnr 379-12, Dnr 227-13, Dnr 277-15, and Dnr 458-15). The study adhered to Reporting Recommendations for Tumor Markers Prognostic Studies (REMARK) criteria (37).

### Tissue Microarray Construction and Immunohistochemistry

Duplicate 1-mm cores from representative tumor regions of formalin-fixed paraffin-embedded tissue blocks were collected from surgical specimens and assembled in a TMA block using a semi-automated tissue array device (Beecher Instruments Inc., Sun Prairie, WI). The TMA blocks were stored at room temperature before sectioning, and the 4-μm thick TMA sections were kept at -20°C until immunohistochemical staining. The sections were automatically deparaffinized before pretreatment using the PT Link system (DAKO, Glostrup, Denmark).

Immunohistochemistry was performed using the Autostainer Plus from DAKO with the EnVision FLEX high-pH kit, according to the manufacturer’s instructions (DAKO, Glostrup, Denmark). A rabbit polyclonal antibody against the AhR, diluted 1:1000 (BML-SA550, Enzo Life Sciences, Farmingdale, NY) was used. The characteristics of the AhR antibody have been reported elsewhere (22, 30). Sections were also stained with a mouse monoclonal antibody to aromatase (clone 677, provided by professor D. Edwards) at 1.9 mg/ml, diluted 1:250, for 1 h at room temperature followed by EnVision FLEX high-pH kit, in an Autostainer Plus, according to the manufacturer’s instructions. The antibody has previously been thoroughly validated for the detection of aromatase by immunohistochemistry (38, 39). Human placenta was used as a positive control for aromatase.

AhR staining was evaluated by two independent observers (SBj and AB), without knowledge of tumor characteristics and patient information. In case of discrepancy, a re-examination was done until consensus was reached or a senior pathologist (KJ) was consulted. Scoring included cytoplasmic staining intensity score (AhR^cyt^); negative, weak, moderate, strong, and nuclear staining (AhR^nuc^); negative, positive. AhR^cyt^ was negative in only 12 tumors (1.3%), and this group was therefore combined with the group with weak staining (n=171) to form a category denoted as low (n=183), and the groups with moderate (n=413) and strong (n=324) staining were combined into a group with high intensity (n=737), thus creating a dichotomized variable for the statistical analyses. In the case of several different AhR^cyt^ intensities within the same tumor cores, the highest intensity with a fraction of >20% of the invasive tumor cells was selected.

Similarly, intratumoral aromatase staining was evaluated by two independent observers (ES and HT), and KJ was consulted in case of discrepancy. Only cytoplasmic staining of aromatase in invasive cells was evaluated. Each tumor was assigned an intensity score (negative, weak, moderate, strong) and a percentage of stained cells. Since intratumoral aromatase was positive in at least 1% of the cells in only 69 cases (8.5%), a dichotomized variable (negative, positive) was used for all analyses.

For both markers, duplicate cores were evaluated jointly. In the case of bilateral tumors (n=17), 12 had an evaluable AhR staining. AhR^cyt^ staining differed on the contralateral side in two cases, but both remained in the same category. AhR^nuc^ staining also differed in two cases, and these changed category. Intratumoral aromatase did not differ in any of the bilateral cases. Data from the tumor on the side with the highest AhR level were used, and all tumor characteristics were taken from the corresponding side. Since only two cases differed, no sensitivity analysis was performed.

### Genotyping

Genomic DNA was obtained for buffy coats from the patients’ leukocyte portion of frozen peripheral blood using the Wizard Genomic DNA Purification Kit (Promega, Madison, USA). Genotyping was performed at the Region Skåne Competence Centre (RSKC Malmö), Skåne University Hospital, Malmö, Sweden, according to the manufacturer’s instructions with reagents included in the iPLEX™ genotyping kit (Sequenom, Inc., San Diego, CA, USA) and the software and equipment in the MassARRAY^®^ platform (Sequenom, Inc., San Diego, CA, USA). The functional *AhR* SNP^Arg554Lys^ (rs2066853) and the *CYP19A1* SNPs (rs10046), (rs4646), Aro1 (rs4775936), and Aro2 (rs10459592) were analyzed. In case of missing values for *CYP19A1*, most could be imputed based on the other SNPs as previously described (3). Haplotypes of *CYP19A1* were constructed by cross-tabulation of the genotypes of the *CYP19A1* SNPs, which resulted in nine haplotypes assembled into four diplotypes and a combined group of rare diplotypes (<10%) as previously described (3). Genotyping was performed in 2008 and was only available for patients included between 2002 and 2008 (n=576).

### Statistics

All statistical analyses were performed using SPSS software version 26 (IBM Corp, Armonk, NY, USA). Variables were dichotomized as follows: age (≥50 years), where age <50 years was used as a proxy for premenopausal status, BMI (≥25 kg/m^2^), waist circumference (≥80 cm), WHR (>0.85), breast volume (≥850 ml), nulliparous, ever use of oral contraceptives (OCs), ever use of menopausal hormone therapy (MHT), coffee intake (≥2 cups/day), current smoker before surgery, alcohol abstainer, adjuvant treatments (chemotherapy, radiotherapy, tamoxifen, AI, and/or herceptin) before any breast cancer event, last follow-up, or death.

Tumor characteristics included invasive pathologic tumor size (≤20 mm, >20 mm, or skin or muscular involvement), any ALNI, histological grade (grade I–III), hormone receptor status (ER, PR), HER2 amplification, and/or triple negativity.

Patient and tumor characteristics were analyzed in relation to AhR levels (AhR^cyt^; high versus low, AhR^nuc^; positive *versus* negative) and intratumoral aromatase expression (positive *versus* negative). Chi-Square tests were used for dichotomous variables, and test for trends was assessed by linear-by-linear association tests. Correlations between staining intensity and time between surgery and staining (years) were calculated with Spearman’s Rho (R_s_). Staining intensity of AhR^cyt^ and AhR^nuc^ was negatively correlated with the time between surgery and staining (*P*<0.0001). Therefore, logistic regression models with patient and tumor characteristics, as well as other analyses, were performed with adjustments for the time between surgery and staining for AhR^cyt^ and AhR^nuc^. Staining of intratumoral aromatase was not correlated with the time between surgery and staining (*P*=0.76).

The impact of AhR^cyt^, AhR^nuc^, and intratumoral aromatase on RFS and DMFS was estimated with Kaplan-Meier curves and assessed with the LogRank test. Groups were formed according to the expression of AhR in different compartments as follows: group 1, high AhR^cyt^ and negative AhR^nuc^; group 2, high AhR^cyt^ and positive AhR^nuc^; group 3, low AhR^cyt^ and positive AhR^nuc^; and group 4, low AhR^cyt^ and negative AhR^nuc^.

Cox regression models were used for multivariable analyses providing hazard ratios (HRs) with 95% confidence intervals (CI). Adjustments were performed in two different models: Model 1, the time between surgery and staining (years; continuous), age (years; continuous), BMI ≥25 kg/m^2^, and tumor characteristics (tumor size >20 mm or skin/muscular involvement irrespective of size, histological grade III, ER status); and Model 2: model 1 with the addition of preoperative smoking, alcohol abstention, and adjuvant treatments (chemotherapy, radiotherapy, tamoxifen, and AI). When comparing four groups of AhR levels in different cellular compartments, as previously described, group 1 (high AhR^cyt^ and negative AhR^nuc^) was used as a reference since it was the largest group.

To examine whether there were any effect modifications by age ≥50 years at inclusion, preoperative BMI ≥25 kg/m^2^, WHR >0.85, tumor characteristics (tumor size >20 mm or skin/muscular involvement, any ALNI, histological grade III, or ER status), preoperative smoking, alcohol abstention, adjuvant treatments (chemotherapy, radiotherapy, tamoxifen and AI) on the associations between AhR^cyt^ and AhR^nuc^ and prognosis, multiplicative interaction variables between these factors and the categories of AhR^cyt^ and AhR^nuc^ were calculated. Interaction analyses were adjusted according to model 1.

Power calculations including 900 patients, of which 20% had low AhR^cyt^ levels and 33% had positive AhR^nuc^ status, with an accrual interval of 10 years and additional follow-up time of seven years, 80% power, and *α* of 0.05, showed that with a mean survival time of nine years it was possible to detect true HRs of ≤0.75 or ≥1.38 for AhR^cyt^ and of ≤0.78 or ≥1.31 for AhR^nuc^. Power calculations, including 804 patients, of which 8.3% had intratumoral aromatase, showed that it was possible to detect true HRs of ≤0.65 or ≥1.66. The power calculations were performed with the PS Power and Sample Size Calculation Program, version 3.1.2 (40). All *P*-values were two-sided, and each *P*-value should be interpreted as the level of evidence against each null hypothesis. Since this is an exploratory study, nominal *P*-values are presented without adjustments for multiple testing (41).

## Results

### Patients Characteristics

Patient characteristics are presented in **Table 1**. The median age at inclusion was 61 years (range, 24–99 years). All *P*-values were adjusted for the time between surgery and staining in AhR analyses. Patients with high AhR^cyt^ levels (n=737/920) had somewhat larger WHR (*P*_adj_=0.046). Positive AhR^nuc^ status (n=292/920) was associated with alcohol abstention (*P*_adj_=0.046). Otherwise, patient characteristics were similar between patients with different levels of AhR^cyt^ and AhR^nuc^. Patients with positive intratumoral aromatase (n=69/816) were younger (*P*=0.001) and more likely to be nulliparous (*P*=0.003) than those with negative status.

**Table 1:** Patient characteristics at inclusion in relation to AhR^cyt^, AhR^nuc,^ and aromatase levels.

	All		AhR^cyt^ (n=920)		AhR^nuc^ (n=920)		Intratumoral aromatase (n=816)	
	patients	Missing	Low	High	Positive	Negative	Positive	Negative
			intensity	intensity				
	(n=1,018)		n=183 (19.9%)	n=737 (80.1%)	n=292 (31.7%)	n=628 (68.3%)	n=69 (8.5%)	n=747 (91.5%)
	Number (%)		Number (%)	Number (%)	Number (%)	Number (%)	Number (%)	Number (%)
**Age ≥50 years**	816 (80.2)	0	147 (80.3)	601 (81.5)	236 (80.8)	512 (81.5)	45 (65.2)	609 (81.5)
**BMI ≥25 kg/m^2^**	503 (50.8)	28	89 (50.6)	368 (51.3)	148 (51.4)	309 (51.0)	34 (52.3)	354 (48.8)
**Waist circumference ≥ 80 cm**	731 (74.6)	38	132 (76.3)	534 (74.9)	220 (77.7)	446 (74.0)	44 (67.7)	541 (75.2)
**Waist-hip ratio >0.85**	519 (53.0)	38	94 (54.3)	379 (53.2)	165 (58.3)	308 (51.1)	30 (46.2)	383 (53.3)
Total breast volume ≥ 850 ml *	492 (57.3)	160	96 (63.6)	352 (56.1)	133 (52.8)	315 (52.8)	32 (56.1)	359 (56.7)
**Parous**	896 (88.0)	0	158 (86.3)	650 (88.2)	257 (88.0)	551 (87.7)	53 (76.8)	664 (88.9)
**Ever use of oral contraceptives**	722 (71.0)	1	127 (69.4)	521 (70.8)	214 (73.3)	434 (69.2)	50 (72.5)	529 (70.9)
**Ever use of MHT**	447 (44.0)	3	77 (42.1)	332 (45.2)	122 (41.9)	287 (45.8)	24 (34.8)	332 (44.6)
**Coffee intake ≥ 2 cups/day**	824 (80.9)	0	152 (83.1)	591 (80.2)	232 (79.5)	511 (81.4)	54 (78.3)	602 (80.6)
**Current smoker prior to surgery**	206 (20.3)	2	35 (19.1)	150 (20.4)	50 (17.2)	135 (21.5)	11 (15.9)	147 (19.7)
**Alcohol abstainer**	106 (10.4)	2	26 (14.2)	70 (9.6)	22 (7.7)	74 (11.8)	7 (10.1)	82 (11.1)
***AhR***								
**Arg554Lys (rs2066853)**		442						
** G/G**	465 (80.7)		104 (80.6)	313 (80.3)	72 (87.8)	345 (78.9)	29 (74.4)	347 (82.0)
** G/A**	103 (17.9)		22 (17.1)	74 (19.0)	10 (12.2)	86 (19.7)	10 (25.6)	71 (16.8)
** A/A**	8 (1.4)		3 (2.3)	3 (0.8)	0 (0.0)	6 (1.4)	0 (0.0)	5 (1.2)
***CYP19A1* diplotypes**		443						
**(rs4646, rs10046, rs4775936, rs10459592)**								
** CCCT_CTTG**	97 (16.9)		18 (14.0)	70 (18.0)	16 (19.5)	72 (16.5)	9 (23.1)	70 (16.6)
** CTTG_CTTG**	134 (23.3)		31 (24.0)	91 (23.4)	24 (29.3)	98 (22.5)	8 (20.5)	97 (23.0)
** CTTG_ACCG**	61 (10.6)		14 (10.9)	43 (11.1)	12 (14.6)	45 (10.3)	6 (15.4)	43 (10.2)
** CTTG_ACCT**	93 (16.2)		20 (15.5)	61 (15.7)	12 (14.6)	69 (15.8)	3 (7.7)	66 (15.6)
** Rare**	190 (33.0)		46 (35.7)	124 (31.9)	18 (22.0)	152 (34.9)	13 (33.3)	146 (34.6)

*breast volume was not analyzed for women with previous breast surgeries.

BMI, body mass index; MHT, menopausal hormonal therapy.

### Tumor Characteristics

Tumor characteristics and clinical data are presented in **Table 2**. AhR^cyt^ levels and AhR^nuc^ status were positively correlated (r_s_=0.22, *P*<0.0001; **Figure 1**). High AhR^cyt^ levels were associated with several favorable tumor characteristics, such as lower histological grade, ER^+^ and PR^+^ status, and lower frequency of triple-negative tumors (all adjusted *Ps ≤* 0.0004). In contrast, positive AhR^nuc^ status was not associated with tumor characteristics apart from the lower frequency of PR^+^ tumors (*P*_adj_=0.036). The highest frequency of ER^+^ tumors (92.7%) was found in patients with high AhR^cyt^ and negative AhR^nuc^ status, while the lowest frequency of ER^+^ tumors (75.0%) was observed in tumors with low AhR^cyt^ and positive AhR^nuc^ status (*P*<0.0001).

**Table 2:** Tumor characteristics, treatments, and events in relation to AhR^cyt^, AhR^nuc^, and intratumoral aromatase levels.

	All		AhR^cyt^ (n=920)		AhR^nuc^ (n=920)		Intratumoral aromatase (n=816)	
	patients	Missing	Low	High	Negative	Positive	Negative	Positive
			intensity	intensity				
	n=1,018		n=183 (19.9%)	n=737 (80.1%)	n=628 (68.3%)	n=292 (31.7%)	n=747 (91.5%)	n=69 (8.5%)
	Number (%)		Number (%)	Number (%)	Number (%)	Number (%)	Number (%)	Number (%)
**Invasive tumor size**		0						
1 > 20 mm	740 (72.7)		126 (68.9)	534 (72.5)	449 (71.5)	211 (72.3)	538 (72.0)	51 (73.9)
> 21 mm	278 (27.3)		57 (31.1)	203 (72.5)	179 (28.5)	81 (27.7)	209 (28.0)	18 (26.1)
or skin/muscular involvement								
**Axillary nodal involvement**		2						
Negative	626 (61.6)		104 (57.1)	452 (61.4)	370 (59.1)	186 (63.7)	446 (59.9)	43 (62.3)
Positive	390 (38.4)		78 (42.9)	284 (38.6)	256 (40.9)	106 (36.3)	299 (40.1)	26 (37.7)
**Histological grade**		1						
I	254 (25.0)		22 (12.1)	199 (27.0)	162 (25.8)	59 (20.2)	171 (22.9)	6 (8.7)
II	506 (49.8)		97 (53.3)	365 (49.5)	320 (51.0)	142 (48.6)	382 (51.1)	30 (43.5)
III	257 (25.3)		63 (34.6)	173 (23.5)	145 (23.1)	91 (31.2)	194 (26.0)	33 (47.8)
**Hormone receptor status**		1						
ER+	894 (87.9)		143 (78.1)	665 (90.4)	559 (89.0)	249 (85.6)	668 (89.5)	44 (63.8)
PR+	721 (70.9)		109 (59.6)	544 (73.9)	455 (72.5)	198 (68.0)	540 (72.4)	33 (47.8)
HER2+	110 (11.5)	63	17 (9.9)	78 (11.0)	65 (11.0)	30 (10.4)	80 (11.0)	10 (14.5)
Triple negative	74 (7.3)	7	28 (15.6)	42 (5.7)	40 (6.4)	30 (10.3)	48 (6.4)	17 (24.6)
**AhR localisation**								
Cytoplasmatic		98						
Low	183 (19.9)		183 (100)	─	163 (26.0)	20 (6.8)	154 (21.1)	9 (13.2)
High	737 (80.1)		─	737 (100)	465 (74.0)	272 (93.2)	576 (78.9)	59 (86.8)
Nuclear positive	292 (31.7)	98	20 (10.9)	272 (36.9)	─	292 (100)	226 (31.0)	25 (36.8)
Intratumoral aromatase positive	69 (8.5)	202	9 (5.5)	59 (9.3)	43 (7.9)	25 (10.0)	─	69 (100)
**Treatments by last follow-up prior to any event^a^**								
Ever use of chemotherapy	259 (25.4)	0	57 (31.1)	176 (23.9)	145 (23.1)	88 (30.1)	188 (25.2)	30 (43.5)
Ever use of radiation therapy	644 (63.3)	0	113 (61.7)	471 (63.9)	393 (62.6)	191 (65.4)	488 (65.3)	40 (58.0)
ER+ tumors only								
Ever use of tamoxifen	572 (64.0)	0	109 (76.2)	422 (63.5)	356 (63.7)	175 (70.3)	440 (65.9)	28 (63.6)
Ever use of AI	371 (41.5)	0	64 (44.8)	279 (42.0)	236 (42.2)	107 (43.0)	289 (43.3)	19 (43.2)
HER2 amplified								
Ever use of trastuzumab*	71 (64.5)	0	9 (52.9)	52 (66.7)	37 (56.9)	24 (80.0)	50 (62.5)	6 (60.0)
**Type of event**								
Any breast cancer event	195 (19.2)	0	46 (25.1)	132 (17.9)	134 (21.3)	44 (15.1)	154 (20.6)	15 (21.7)
Distant metastasis	122 (12.0)	0	31 (16.9)	82 (11.1)	88 (14.0)	25 (8.6)	95 (12.7)	12 (17.4)
Death	188 (18.5)	0	49 (26.8)	127 (17.2)	134 (21.3)	42 (14.4)	142 (19.0)	17 (24.6)

^a^Most patients received more than one type of treatment.

ER, estrogen receptor; PR, progesterone receptor.

*One additional patient had HER2 positive tumor on the contralateral side and therefore also received trastuzumab.

A lower proportion of patients with high compared with low AhR^cyt^ levels received adjuvant chemotherapy (*P*_adj_=0.0004). Adjuvant treatment with tamoxifen was associated with low AhR^cyt^ levels (*P*=0.010) and positive AhR^nuc^ status (*P*=0.0032) while radiotherapy or AI treatment showed no association with either AhR^cyt^ or AhR^nuc^ status.

Intratumoral aromatase status was not correlated with either AhR^cyt^ or AhR^nuc^ status (see **Figure 1**). Positive intratumoral aromatase status was strongly associated with ER^–^ and PR^–^ status, triple negativity, and higher histological grade (all *P*s *≤* 0.0002). In line with this, positive intratumoral aromatase status was associated with adjuvant chemotherapy (*P*=0.001) but not with adjuvant endocrine therapy or radiotherapy.

### Tumors With Non-Evaluable Staining

Patients whose tumors could not be evaluated for AhR (n=98) were somewhat younger than included patients and had smaller tumors and less ALNI than patients with evaluable AhR. Also, they had more HER2^+^ tumors, no difference in ER and PR status, but received less adjuvant endocrine therapy. Patients whose tumors could not be evaluated for intratumoral aromatase expression (n=202) had less ALNI and lower histological grade, no difference in hormone receptors, but received less endocrine therapy than patients with evaluable aromatase expression.

### *AhR* and *CYP19A1* Genotypes and AhR and Intratumoral Aromatase Status

There was no correlation between the *AhR* ^Arg554Lys^ and any of the *CYP19A1* genotypes. The functional *AhR* ^Arg554Lys^ genotype (n=576) was not clearly associated with either AhR^cyt^ or AhR^nuc^ levels (both *P*s≥0.088). None of the *CYP19A1* genotypes or diplotypes (n=575) were associated with intratumoral aromatase. However, the *CYP19A1* diplotypes were associated with AhR^nuc^ status (*P*=0.033, 4 degrees of freedom (d.f.)). Rare *CYP19A1* diplotypes were more common in patients with positive AhR^nuc^ tumors.

### Follow-up and Events

Patients received follow-up questionnaires for up to 15 years from inclusion. For the 920 patients with invasive cancer included in the AhR survival analyses, 660 patients were still at risk by the end of follow-up, and for these patients, the median follow-up time was 8.7 years (IQR 6.9–11.0). During the follow-up time, breast cancer events occurred in 178 patients, of whom 113 had distant metastases. Also, 176 patients died, and 94 of these had a prior breast cancer event.

### Intratumoral AhR Levels in Relation to Prognosis

**Figure 2:**
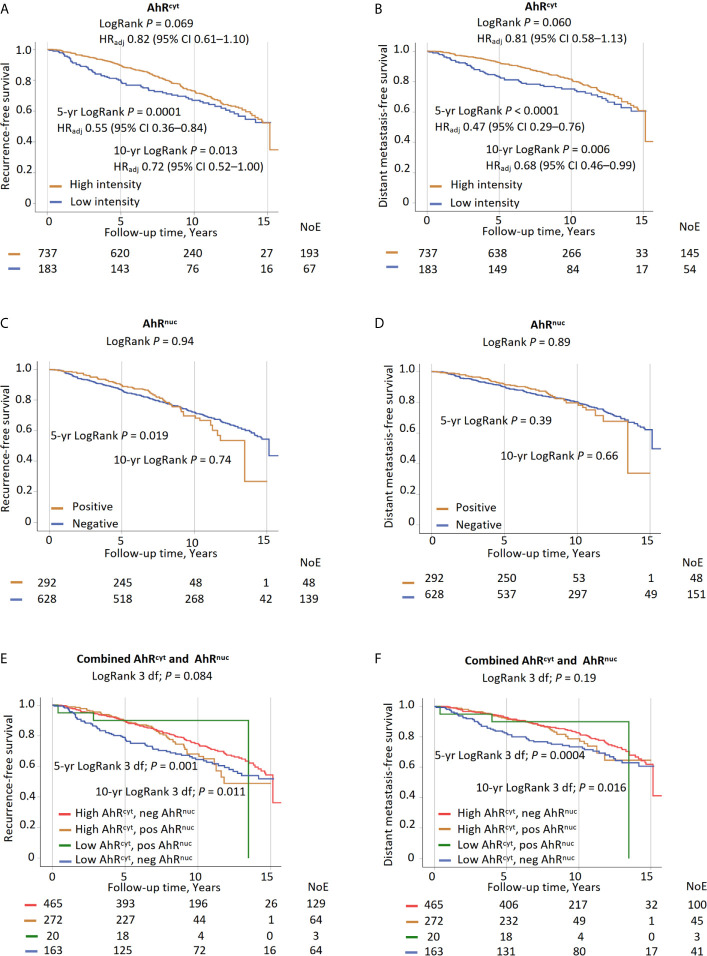
High AhR^cyt^ levels compared to low AhR^cyt^ in relation to 5-year, 10-year, and the entire follow-up: **(A)** recurrence-free survival and **(B)** distant metastasis-free survival. Positive AhR^nuc^ levels compared to negative AhR^nuc^ status in relation to 5-year, 10-year, and the entire follow-up **(C)** recurrence-free survival and **(D)** distant metastasis-free survival. Associations (3 d.f.) between four groups of AhR^cyt^ levels and AhR^nuc^ status (high AhR^cyt^ and positive AhR^nuc^; high AhR^cyt^ and negative AhR^nuc^; low AhR^cyt^ and positive AhR^nuc^; low AhR^cyt^ and negative AhR^nuc^) and 5-year, 10-year and the entire follow-up **(E)** recurrence-free survival and **(F)** distant metastasis-free survival.

High AhR^cyt^ levels were associated with a better 10-year prognosis in terms of both RFS and DMFS in univariable analyses. The curves merged by the 15-year follow-up (**Figures 2A, B**). In multivariable analyses, high AhR^cyt^ levels were associated with approximately half the risk of an event by the 5-year follow-up, HR_adj_ 0.55 (95% CI 0.36–0.84), and 5-year RFS of patients with high AhR^cyt^ levels was 89.6% compared with 79.6% for patients with low levels. The association became weaker by the 10-year follow-up, HR_adj_ 0.72 (95% CI 0.52–1.00). The results were mainly the same for DMFS, with 5-year DMFS of 92.5% in patients with high AhR^cyt^ levels compared with 82.9% in patients with low levels. In contrast, AhR^nuc^ was not associated with RFS or DMFS (**Figures 2C, D**).

Patients were divided into four groups depending on the subcellular localization of AhR (**Table 3**). By the 10-year follow-up, patients with low AhR^cyt^ and negative AhR^nuc^ had the poorest prognosis, both in terms of RFS, HR_adj_ 1.65 (95% CI 1.16–2.33), and DMFS, HR_adj_ 1.69 (95% CI 1.12–2.55). Beyond that, no clear associations were found (**Figures 2E, F**). When only considering patients with more than five years of follow-up, those with high AhR^cyt^ and positive AhR^nuc^ had a tendency towards more late recurrences.

**Table 3:** Multivariable models with crude and adjusted HR (95% CIs) for combined AhR^cyt^ and AhR^nuc^ in relation to RFS and DMFS for 5-yr, 10-yr, and the entire follow-up.

Recurrence-free survival																				
									Model 1				Model 2				Model 3			
		Total		Events	Crude			*P*-value	Adjusted			*P*-value	Adjusted			*P*-value	Adjusted			*P*-value
Combined AhR^cyt^ & AhR^nuc^		**n**		**n**	**HR 5-yr**	**(95% CI)**			**HR 5-yr**	**(95% CI)**		** **	**HR 5-yr**	**(95% CI)**		** **	**HR 5-yr**	**(95% CI)**		** **
High AhR^cyt^ & neg AhR^nuc^		465		47	Ref			0.001	Ref			0.018	Ref			0.085	Ref			0.020
High AhR^cyt^ & pos AhR^nuc^		272		27	0.99	0.61	1.58		1.27	0.76	2.11		1.14	0.68	1.91		1.29	0.76	2.20	
Low AhR^cyt^ & pos AhR^nuc^		20		2	0.98	0.24	4.04		1.20	0.29	4.98		0.85	0.20	3.56		1.00	0.24	4.23	
Low AhR^cyt^ & neg AhR^nuc^		163		35	2.26	1.46	3.50		2.04	1.31	3.19		1.79	1.13	2.83		2.10	1.32	3.34	
Combined AhR^cyt^ & AhR^nuc^		**n**		**n**	**HR 10-yr**	**(95% CI)**		** **	**HR 10-yr**	**(95% CI)**		** **	**HR 10-yr**	**(95% CI)**		** **	**HR 10-yr**	**(95% CI)**		** **
High AhR^cyt^ & neg AhR^nuc^		465		99	Ref			0.013	Ref			0.025	Ref			0.052	Ref			0.018
High AhR^cyt^ & pos AhR^nuc^		272		58	1.16	0.83	1.60		1.28	0.90	1.81		1.25	0.88	1.79		1.37	0.95	1.97	
Low AhR^cyt^ & pos AhR^nuc^		20		2	0.50	0.12	2.03		0.54	0.13	2.21		0.47	0.11	1.91		0.51	0.13	2.11	
Low AhR^cyt^ & neg AhR^nuc^		163		54	1.67	1.20	2.33		1.61	1.15	2.25		1.52	1.08	2.15		1.65	1.16	2.33	
Combined AhR^cyt^ & AhR^nuc^		**n**		**n**	**HR**	**(95% CI)**		** **	**HR**	**(95% CI)**		** **	**HR**	**(95% CI)**		** **	**HR**	**(95% CI)**		
High AhR^cyt^ & neg AhR^nuc^		465		129	Ref			0.087	Ref			0.10	Ref			0.16	Ref			0.062
High AhR^cyt^ & pos AhR^nuc^		272		64	1.16	0.86	1.58		1.27	0.91	1.75		1.24	0.89	1.74		1.35	0.96	1.90	
Low AhR^cyt^ & pos AhR^nuc^		20		3	0.64	0.20	2.00		0.68	0.21	2.14		0.58	0.18	1.83		0.59	0.18	1.88	
Low AhR^cyt^ & neg AhR^nuc^		163		64	1.43	1.06	1.94		1.39	1.03	1.88		1.32	0.96	1.80		1.41	1.03	1.93	
**Distant metastasis-free survival**																				
		** **			** **	** **	** **	** **	**Model 1**		** **	** **	**Model 2**		** **	** **	**Model 3**		** **	** **
		**Total**	**Events**		**Crude**		** **	***P*-value**	**Adjusted**		** **	***P*-value**	**Adjusted**		** **	***P*-value**	**Adjusted**		** **	***P*-value**
Combined AhR^cyt^ & AhR^nuc^		**n**	**n**		**HR 5-yr**	**(95% CI)**		** **	**HR 5-yr**	**(95% CI)**		** **	**HR 5-yr**	**(95% CI)**		** **	**HR 5-yr**	**(95% CI)**		** **
High AhR^cyt^ & neg AhR^nuc^		465	32		Ref			0.001	Ref			0.005	Ref			0.047	Ref			0.015
High AhR^cyt^ & pos AhR^nuc^		272	21		1.14	0.66	1.97		1.36	0.75	2.44		1.18	0.65	2.16		1.27	0.68	2.38	
Low AhR^cyt^ & pos AhR^nuc^		20	2		1.44	0.35	6.01		1.66	0.39	6.99		1.22	0.29	5.20		1.46	0.34	6.32	
Low AhR^cyt^ & neg AhR^nuc^		163	29		2.70	1.64	4.47		2.51	1.51	4.18		2.12	1.25	3.60		2.42	1.41	4.16	
Combined AhR^cyt^ & AhR^nuc^		**n**	**n**		**HR 10-yr**	**(95% CI)**		** **	**HR 10-yr**	**(95% CI)**		** **	**HR 10-yr**	**(95% CI)**		** **	**HR 10-yr**	**(95% CI)**		** **
High AhR^cyt^ & neg AhR^nuc^		465	68		Ref			0.018	Ref			0.036	Ref			0.12	Ref			0.082
High AhR^cyt^ & pos AhR^nuc^		272	40		1.14	0.77	1.69		1.23	0.81	1.87		1.19	0.78	1.83		1.25	0.80	1.94	
Low AhR^cyt^ & pos AhR^nuc^		20	2		0.73	0.18	2.98		0.78	0.19	3.18		0.67	0.16	2.76		0.73	0.18	3.04	
Low AhR^cyt^ & neg AhR^nuc^		163	41		1.82	1.24	2.69		1.77	1.19	2.62		1.60	1.07	2.41		1.69	1.12	2.56	
Combined AhR^cyt^ & AhR^nuc^		**n**	**n**		**HR**	**(95% CI)**		** **	**HR**	**(95% CI)**		** **	**HR**	**(95% CI)**		** **	**HR**	**(95% CI)**		** **
High AhR^cyt^ & neg AhR^nuc^		465	100		Ref			0.19	Ref			0.25	Ref			0.48	Ref			0.33
High AhR^cyt^ & pos AhR^nuc^		272	45		1.10	0.77	1.58		1.18	0.80	1.73		1.15	0.77	1.70		1.21	0.81	1.81	
Low AhR^cyt^ & pos AhR^nuc^		20	3		0.87	0.28	2.75		0.91	0.29	2.89		0.77	0.24	2.45		0.77	0.24	2.49	
Low AhR^cyt^ & neg AhR^nuc^		163	51		1.44	1.03	2.02		1.41	1.00	1.98		1.29	0.90	1.83		1.36	0.95	1.94	

Model 1: Time between surgery and staining

Model 2: Adjusted for model 1 + age, BMI ≥25 kg/m^2^, tumor size, node status, grade III, ER status. Missing data for 30 patients for at least one variable.

Model 3: Adjusted for model 1+2 + preoperative smoking, alkohol abstention and adjuvant treatments. Missing data for 34 patients for at least one variable.

### Effect Modifications Between AhR^cyt^ Levels and Endocrine Therapy on Prognosis

Formal interaction analyses were conducted to study effect modifications between patient and tumor characteristics, treatments, and the AhR^cyt^ and AhR^nuc^ on prognosis. No effect modifications were found between AhR^cyt^ levels and patients or tumor characteristics. However, an interaction was found between AhR^cyt^ and any AI treatment (*P*_interaction_=0.030). No interactions were found between AhR^cyt^ and chemotherapy, radiotherapy, or tamoxifen. Several of the patients who received any AI treatment also had received tamoxifen (switch-therapy). Therefore, three new variables were formed; tamoxifen only, AI only, and switch-therapy.

**Figure 3:**
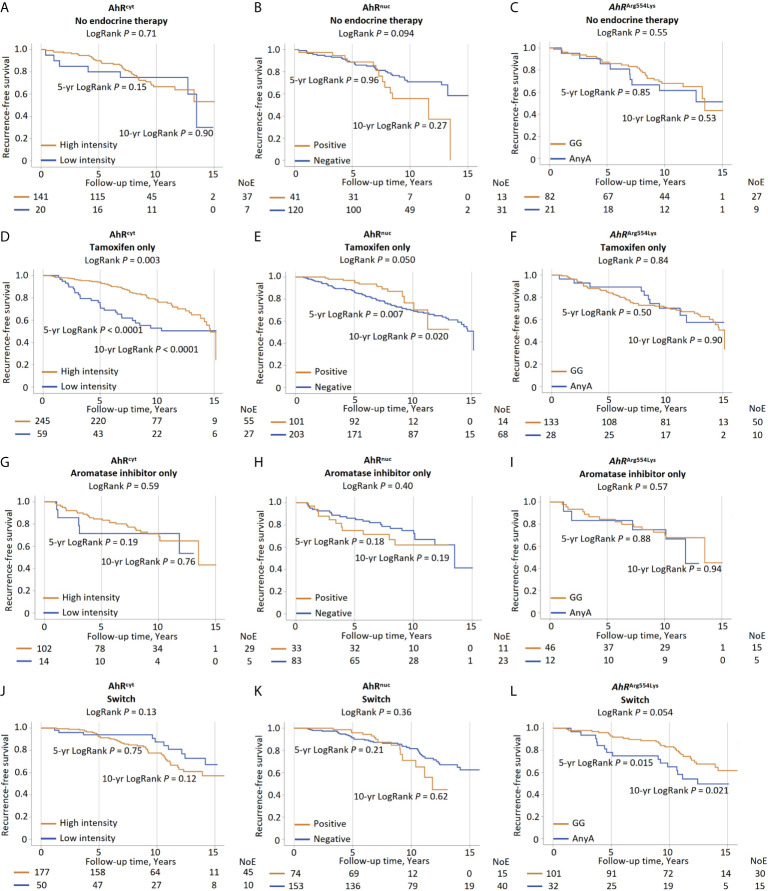
High AhR^cyt^ compared to low AhR^cyt^ levels in patients with ER^+^ tumors in relation to 5-year, 10-year and the entire follow-up recurrence-free survival in patients with **(A)** no endocrine therapy, **(D)** tamoxifen only, **(G)** aromatase inhibitors only, and **(J)** switch-therapy. Positive AhR^nuc^ levels compared to negative AhR^nuc^ status in relation to 5-year, 10-year, and the entire follow-up recurrence-free survival in patients with **(B)** no endocrine therapy, **(E)** tamoxifen only, **(H)** aromatase inhibitors only, and **(K)** switch-therapy. *AhR* ^Arg554Lys^ GG compared any A genotype in relation to 5-year, 10-year and the entire follow-up recurrence-free survival in patients with **(C)** no endocrine therapy, **(F)** tamoxifen only, **(I)** aromatase inhibitors only, and **(L)** switch-therapy.

Interaction analyses with patients with ER^+^ tumors were conducted, including these variables. In this model, the effect modification seemed driven by switch-therapy rather than by AI only (*P*_interaction_=0.82). The prognostic impact of AhR^cyt^ in endocrine treatment groups is shown in **Figures 3A, F, G,** **J**. High AhR^cyt^ levels were strongly associated with good 10-year prognosis in tamoxifen-treated patients, HR_adj_ 0.40 (95% CI 0.23–0.71), 10-year RFS was 78.2% in patients with high AhR^cyt^ levels compared with 52.9% in patients with low levels, while almost an inverse association was seen in patients who received switch-therapy (*P*_interaction_=0.023).

### Effect Modifications Between AhR^nuc^ Status and Clinicopathological Factors on Prognosis

An interaction was found between AhR^nuc^ and age ≥50 years on RFS where younger patients with positive AhR^nuc^ status had a better prognosis compared to patients with negative AhR^nuc^ status, HR_adj_=0.40 (95% CI 0.17–0.99), an association not seen in older patients (*P*_interaction_=0.011). Another interaction between AhR^nuc^ and any use of chemotherapy was found where chemonaïve patients with positive AhR^nuc^ status had a poorer prognosis than patients with negative AhR^nuc^ status, HR_adj_=1.54 (95% CI 1.06–2.25), while no difference was seen in chemotherapy-treated patients (*P*_interaction_=0.008). No interaction was found between AhR^nuc^ and any adjuvant use of radiotherapy, tamoxifen, or AI.

Similarly, we conducted an additional interaction analysis in patients with ER^+^ tumors with tamoxifen only, AI only, and switch-therapy. In this analysis, an interaction was found between AhR^nuc^ and the use of tamoxifen only (*P*_interaction_=0.041) on RFS, but not with AI only or switch-therapy (**Figures 3B, E, H, K**).

### Effect Modifications Between *AhR* ^Arg554Lys^ and Endocrine Therapy on Prognosis

In line with our previously reported findings, switch-treated patients with *AhR* ^Arg554Lys^ (GG) genotype had a better prognosis than any A genotypes, HR_adj_ 0.42 (95% CI 0.22–0.83). This association was strongest during a 10-year follow-up (**Figure 3L**). No clear associations were found in other treatment groups (**Figures 3C, F, I**). However, no significant effect modifications were found between the *AhR* ^Arg554Lys^ genotype and endocrine therapy.

### *AhR* and Intratumoral Aromatase Status in Relation to Prognosis

Overall, intratumoral aromatase was not associated with RFS (LogRank *P*=0.43) or DMFS (LogRank *P*=0.17). Since there were few patients with tumors with positive aromatase status, no further analyses were conducted.

## Discussion

The main findings in this study were that high AhR^cyt^ levels were associated with favorable tumor characteristics and clinical outcomes in primary breast cancer patients, while AhR^nuc^ status was not associated with overall prognosis. The prognostic impact of AhR^cyt^ was substantially modified by the type of endocrine therapy. The largest difference was seen between tamoxifen only and switch-therapy. Likewise, the prognostic impact of *AhR* ^Arg554Lys^ genotype also differed between endocrine treatment groups. AhR^nuc^ status was prognostic in chemonaïve but not in chemotherapy-treated patients. Intratumoral aromatase was associated with several aggressive tumor characteristics but not with AhR levels or prognosis. Neither AhR levels nor intratumoral aromatase status was associated with their respective genotypes.

Associations between favorable tumor characteristics and AhR levels seen in the present study have previously been reported in some (22) but not all (23, 42) studies. In line with our findings, two previous studies reported good prognosis with higher AhR levels (5, 22) but another study reported no association (19). In contrast, a couple of studies suggested associations between high AhR expression and poor prognosis (21, 23). To our knowledge, only one previous study evaluated the impact of AhR^cyt^ and AhR^nuc^ levels separately and found a negative prognostic impact of higher AhR^nuc^ levels in lymph node-negative patients, who are less likely to receive chemotherapy, while AhR^cyt^ levels were not associated with prognosis (23). However, no formal interaction analysis was presented in the paper. Our results do not support their finding since no effect modifications between ALNI and AhR levels on prognosis were found.

Patients with high AhR^cyt^ and negative AhR^nuc^ status had the best prognosis during the entire follow-up period in the present study. Therefore, we hypothesize that it is predominantly the inactivated AhR^cyt^ that confers a good prognosis in breast cancer. Although patients with high AhR^cyt^ and positive AhR^nuc^ status had good initial outcomes during the first five years, when endocrine therapy is given to most patients with ER^+^ tumors, late recurrences were more common in this group. These patients might benefit from extended endocrine therapy. It is possible that a ligand-activated, translocated AhR^nuc^ and the downstream transcription of multiple pathways confers a negative prognostic impact that is only apparent after completion of endocrine therapy. The worst initial outcomes were associated with low AhR^cyt^ and negative AhR^nuc^ status. Since only a handful of patients had positive AhR^nuc^ status and low AhR^cyt^ levels, we could not draw any conclusions regarding this combination.

*In vitro* studies suggest that AhR activation induces intratumoral aromatase, which in turn increases intratumoral estrogen synthesis and proliferation (43, 44) while at the same time inhibiting the ER pathway in breast cancer cells (27, 45, 46). Although no overall correlation was found between intratumoral aromatase status and AhR levels in the present study, an exploratory analysis revealed a marginally positive correlation between intratumoral aromatase and AhR^cyt^ levels in patients with ER^+^ but not ER^–^ tumors (data not shown). However, the number of patients was small, and the level of evidence was low.

Interesting interactions were found between AhR^cyt^ and AhR^nuc^ status and endocrine therapy in relation to RFS. To our knowledge, this is the first study suggesting an interplay between AhR levels and the type of endocrine therapy in breast cancer. Patients with high AhR^cyt^ levels who only received endocrine therapy with tamoxifen had an excellent prognosis during the first 10 years of follow-up, whereas an almost inverse relationship was seen for patients treated with switch-therapy between tamoxifen and AIs. Further, switch-treated patients with the *AhR* ^Arg554Lys^ GG genotype had favorable outcomes compared to patients with any A genotype. No prognostic impact of the *AhR* ^Arg554Lys^ genotype was seen in other endocrine treatment groups. The homozygous AA genotype has been associated with lower mRNA expression of AhR (11), but in the present study, no correlation between genotypes and protein levels were found.

Positive intratumoral aromatase status was only found in 8.5% of the patients in the cohort. This is in contrast with previous findings in a small study using the same monoclonal antibody, where intratumoral aromatase staining was denoted in 17 of 28 tumors (47). However, aromatase can be heterogeneously expressed in the tumor (30, 47) and the use of TMA instead of whole tumor sections in the present study might have yielded an underestimation of the intratumoral aromatase status (48). Intratumoral aromatase status was associated with hormone receptor negativity and other aggressive tumor characteristics but not AhR in the current study. Thus, we could not confirm the previous *in vitro* findings that AhR induces intratumoral aromatase in breast cancer (30), but due to the limited number of aromatase-positive tumors, these findings should be interpreted with caution.

In contrast to our hypothesis that anthropometric factors would influence tumor levels of AhR and aromatase, only a marginal association between WHR and AhR was found. Also, a marginal inverse association with alcohol abstention (49) but not smoking (7) was observed. Younger and nulliparous patients were substantially more likely to have tumors positive for intratumoral aromatase, both factors associated with ER^–^ status (50). ER^–^ status was strongly associated with intratumoral aromatase status in the present study, which is in contrast to a previous report (48). Additional staining for classical AhR targets such as CYP1A1 and CYP1B1 to confirm the activity status of AhR would have been interesting but was outside the scope of this study.

During the inclusion period, more than half of the primary breast cancer patients undergoing breast surgery in Lund were included in the cohort (32). The main reason for non-inclusion was a limited number of research nurses. The included patients did not differ substantially from all breast cancer patients operated in Lund during this period (32). Therefore, the findings can be considered generalizable for breast cancer patients treated at this clinic. However, evaluable TMA cores were slightly more often missing for patients with less advanced tumors than for patients with more advanced tumors, but this differed somewhat between the two markers. Missing evaluable tumor tissue could potentially cause selection bias in the analyses. Prognostic associations for AhR became stronger when HER2 was included in the multivariable models (data not shown). However, HER2 status was more often missing from small tumors with longer follow-up times (36) and inclusion of this marker could bias the analyses. Therefore, HER2 status was not used in the multivariable analyses presented. MHT was not associated with either marker. Since we have previously reported that MHT had no overall prognostic impact in this cohort (51), MHT was not included in the adjustment models. In this observational study, we took commonly used prognosticators into account in the multivariable models to evaluate the independent prognostic value of each marker, but residual confounding remains possible. We also adjusted for the time between surgery and staining since AhR levels were lower the longer the tumors had been stored. This time variable also captures other changes occurring during the period, such as changes in treatments. Cox proportional hazards models were used. However, since not all hazards were proportional, we also divided the follow-up period into 5-year and 10-year follow-ups. Although statistical power was decent in the entire cohort, the number of patients and events in some subgroups were smaller, which led to lower power in these analyses.

## Conclusion

In conclusion, our results suggest that AhR^cyt^ levels are associated with ER^+^ status and other favorable tumor characteristics and prognosis. The prognostic impact of AhR was substantially modified by the type of endocrine therapy where high AhR^cyt^ levels were associated with significantly longer recurrence-free survival during the first 10 years of follow-up among patients who received tamoxifen as only endocrine therapy compared to patients with low AhR^cyt^ levels, whereas an almost inverse relationship was seen in patients treated with switch-therapy. Our findings suggest that both AhR^cyt^ levels and *AhR* ^Arg554Lys^ genotypes merit further study as to whether they can be used to guide the selection of endocrine therapy for patients with ER^+^ breast cancer in the clinical setting. These findings warrant confirmation in an independent cohort, preferably in a randomized clinical trial comparing different regimens of tamoxifen and AIs. They might also guide the selection of breast cancer patients for inclusion in trials with selective AhR modulators.

## Data Availability Statement

The data analyzed in this study is subject to the following licenses/restrictions: Data is not publicly available due to privacy laws. Questions regarding data should be directed to helena.jernstrom@med.lu.se.

## Ethics Statement

The studies involving human participants were reviewed and approved by Lund University Ethics committee. The patients/participants provided their written informed consent to participate in this study.

## Author Contributions

HT and HJ: study design, manuscript preparation, statistical analysis, data analysis, and interpretation. HJ, KI, and SBo: study supervision. HT, ES, SBj, AB, MR, SK, BN, KJ, and HJ: data collection. DE: provision of the anti-aromatase 677 antibody. HT, ES, SBj, AB, MR, SK, DE, BN, KJ, KI, SBo, and HJ: contributed to the manuscript review, critical revision for important intellectual content, and read and approved the final draft for submission. All authors are responsible for the manuscript content.

## Funding

This work was supported by grants from The Swedish Cancer Society (CAN2017/368), the Medical Faculty at Lund University, the Mrs. Berta Kamprad Foundation, the South Swedish Health Care Region (Region Skåne ALF 40620) Region Skåne ST-ALF, and the Skåne University Hospital Fund. Open access funding provided by Lund University. The funding agencies played no role in the study’s design, the collection, analysis, and interpretation of the data, the writing of the manuscript, or in the decision to submit the manuscript for publication.

## Conflict of Interest

The authors declare that the research was conducted in the absence of any commercial or financial relationships that could be construed as a potential conflict of interest.

## Abbreviations

AhR, aryl hydrocarbon receptor; AhR^cyt^, cytoplasmic aryl hydrocarbon receptor; AhR^nuc^, nuclear aryl hydrocarbon receptor; AI, aromatase inhibitor; ALNI, axillary lymph node involvement; ARNT, aryl hydrocarbon receptor nuclear translocator; BC, breast cancer; BMI, body mass index; CI, confidence interval; CYP, cytochrome P450; DFS, disease-free survival; DMFS, disease metastasis-free survival; DNA, deoxyribonucleic acid; ER, estrogen receptor; HER2, human-epidermal growth factor receptor 2; HR, hazard ratio; IGF, insulin-like growth factor; MHT, menopausal hormone therapy; OC, oral contraceptives; PR, progesterone receptor; REMARK, recommendations for Tumor Marker Prognostic Studies; SERM, selective estrogen receptor modulator; TMA, tumor tissue microarray; WHR, waist-to-hip ratio.
